# Functional Dissection of Genes Encoding DNA Polymerases Based on Conditional Mutants in the Heterocyst-Forming Cyanobacterium *Anabaena* PCC 7120

**DOI:** 10.3389/fmicb.2020.01108

**Published:** 2020-06-03

**Authors:** Wei-Yue Xing, Li-rui Xie, Xiaoli Zeng, Yiling Yang, Cheng-Cai Zhang

**Affiliations:** ^1^State Key Laboratory of Freshwater Ecology and Biotechnology, Institute of Hydrobiology, Chinese Academy of Sciences, Wuhan, China; ^2^Key Laboratory of Algal Biology, Institute of Hydrobiology, Chinese Academy of Sciences, Wuhan, China; ^3^University of Chinese Academy of Sciences, Beijing, China; ^4^Institut WUT-AMU, Aix-Marseille Université and Wuhan University of Technology, Wuhan, China

**Keywords:** heterocyst, DNA replication, cell cycle, SOS response, cyanobacteria

## Abstract

The filamentous cyanobacterium *Anabaena* sp. PCC 7120 develops N_2_-fixing heterocyst cells under condition of combined-nitrogen deprivation and constitutes an excellent model for studying cell differentiation. The mechanism of heterocyst development has been extensively investigated and a network of regulating factors has been identified. A few studies have showed that the process of heterocyst differentiation relates with cell cycle events, but further investigation is still required to understand this relationship. In a previous study, we created a conditional mutant of PolI encoding gene, *polA*, by using a CRISPR/Cpf1 gene-editing technique. Here, we were able to create another conditional mutant of a PolIII encoding gene *dnaENI* using a similar strategy and subsequently confirmed the essential roles of both *polA* and *dnaENI* in DNA replication. Further investigation on the phenotype of the mutants showed that lack of PolI caused defects in chromosome segregation and cell division, while lack of DnaENI (PolIII) prevented bulk DNA synthesis, causing significant loss of DNA content. Our findings also suggested the possible existence of a SOS-response like mechanism operating in *Anabaena* PCC 7120. Moreover, we found that heterocyst development was differently affected in the two conditional mutants, with double heterocysts/proheterocysts found in PolI conditional mutant. We further showed that formation of such double heterocysts/proheterocysts are likely caused by the difficulty in nucleoids segregation, resulting delayed, or non-complete closure of the septum between the two daughter cells. This study uncovers a link between DNA replication process and heterocyst differentiation, paving the way for further studies on the relationship between cell cycle and cell development.

## Introduction

Cyanobacteria are some of the most abundant organisms on earth and exhibit tremendous diversities in cell shape, life cycle and cellular metabolism. They perform oxygenic photosynthesis and share a common ancestor with plant chloroplast (McFadden and van Dooren, 2004). Among many cyanobacteria, *Anabaena* (*Nostoc*) sp. strain PCC 7120 (hereafter *Anabaena* PCC 7120) is interesting because of its multicellularity and the presence of different cell types: vegetative cells and heterocysts. When combined nitrogen source is limited, about 5–10% of the vegetative cells are induced to form heterocysts, cells devoted to fix atmospheric nitrogen (Wolk et al., 1994). Heterocysts are morphologically different from vegetative cells, with a thicker cell envelope to limit penetration of oxygen, which is known to be toxic to nitrogenase. The two different types of cells are highly collaborative, i.e., heterocysts depend on vegetative cells for reduced carbon sources and as return, provides vegetative cells with fixed nitrogen (Herrero et al., 2016). In *Anabaena* PCC 7120, heterocysts are distributed semi-regularly along the filaments, interspaced by approximately 10–12 vegetative cells to allow efficient nutrient exchange (Wolk et al., 1994). Heterocyst differentiation is a highly regulated process. NtcA is a global nitrogen regulator that under conditions of nitrogen deprivation, triggers a range of downstream responses important for heterocyst differentiation (Zhang et al., 2006; Herrero et al., 2016). Another central regulatory protein is HetR, a positive regulator required for heterocyst development and pattern formation. Inactivation of HetR blocks heterocyst formation in the absence of combined nitrogen, whereas excess HetR results in the multiple-contiguous heterocysts (Mch) phenotype (Buikema and Haselkorn, 1991; Risser and Callahan, 2008).

So far, little is known about cyanobacterial cell cycle and much of our knowledge comes from the model bacteria *Escherichia coli, Bacillus subtilis*, and *Caulobacter crescentus* (Willis and Huang, 2017). In *E. coli*, high fidelity DNA replication requires two DNA polymerases: DNA Polymerase I (PolI) and DNA polymerase III (PolIII). PolI removes the RNA primers and fills the gaps on both the leading and lagging strands, playing important roles in maturation of Okazaki fragments and DNA repair. PolIII is a holoenzyme consists of many protein subunits, among which DnaE is the one responsible for polymerizing complimentary DNA on both strands. After replication, the two daughter nucleoids need to be properly segregated, in coordination with the subsequent cell division, to ensure that each daughter cell receives one copy of the two chromosomes (Dewachter et al., 2018). Prior to cell division, short filaments of FtsZ protein assemble into a ring-like structure at the mid-cell, landmarking the site for future cell division (Ma et al., 1996). With the help of FtsZ-ring and other auxiliary proteins, a division septum is formed and subsequently split the mother cell into two daughter cells (Egan and Vollmer, 2013). The Min system and the nucleoid occlusion (Noc) system are required to place FtsZ-ring at the right place in the right time, thereby preventing the chromosomes from being guillotined by the division septum before they are completely segregated (Ortiz et al., 2016). Despite sharing many cell cycle genes with the model bacteria (Miyagishima et al., 2005), studies in cyanobacterial cell cycle suggest a different scenario may exist. First of all, many cyanobacteria contain multiple copies of chromosomal DNA in a single cell, different from that in *E. coli*. Second, the replication initiation factor DnaA is not essential for DNA replication in some cyanobacteria, at least not in *Synechocystis* PCC 6803 and *Anabaena* PCC 7120 (Ohbayashi et al., 2016), suggesting the presence of an alternative mechanism independent of DnaA/*oriC* for the initiation of DNA replication in these cyanobacteria. Third, the event of DNA replication has been found active throughout the cell cycle progression, i.e., no apparent chromosome replication period (C-period) was observed as seen in *E. coli* during slow growth (Sakr et al., 2006b). In addition, studies in *Synechocystis* found that the multiple chromosomes are segregated randomly and passively through the formation of septum that closes just before the complete segregation of chromosomes (Schneider et al., 2007), suggesting a different mechanism of chromosome segregation from that of *E. coli*. In the case of *Anabaena* PCC 7120, previous studies suggested that heterocyst formation may be subjected to the cell cycle regulation, while the cell cycle is also under the developmental control (Sakr et al., 2006a, b). For example, several mutants (*hetF*, *patU3*) affected in heterocyst differentiation display a certain phenotype in cell size or cell division (Zhang et al., 2007; Risser and Callahan, 2008) and the PatA protein is shown to be localized at the mid-cell position (Young-Robbins et al., 2010); on the other hand, *ftsZ* is downregulated at both transcriptional and posttranscriptional levels during heterocyst differentiation (Sakr et al., 2006a; He and Xu, 2010) and the inhibition of cell division blocks heterocyst differentiation (Sakr et al., 2006a). Another study suggests that the ratio of DnaA/*oriC* is important for heterocyst differentiation (Ohbayashi et al., 2016). However, the detailed mechanism on the connection between the cell cycle events and the process of heterocyst differentiation remains unexplored.

As one of the major cell cycle check-point mechanism in prokaryotic organisms, SOS reponse plays an important role in arresting cell division when DNA replication is interrupted. Bacteria are constantly challenged by internal or external DNA-damaging factors, such as reactive oxygen species (ROS) from cellular metabolism and UV radiation from sunlight (Sinha and Häder, 2002). To cope with the sudden increase of DNA damage, many bacteria employ SOS responses to arrest cell division temporally in order to perform error-free or error-prone repair before proceeding to the next stage of the cell cycle (Sassanfar and Roberts, 1990). A typical bacterial SOS response involves activating a class of genes (*din* genes), among which *lexA* and *recA* are the most important regulators. LexA repressor binds to SOS operator sequences to prevent SOS-related gene expression under normal conditions. When DNA damage occurs, RecA binds to single stranded DNA and changes conformation, causing autocleavage of LexA, and therefore allowing transcription of the corresponding SOS genes (Walker, 1984; Patel et al., 2010). Key genes related to the SOS response have been found in the genomes of many cyanobacteria (Mazón et al., 2004; Li et al., 2010). Recent studies showed that in *Anabaena* PCC 7120, the autocleavage activity of LexA is observed only at alkaline pH and is independent of RecA (Kumar et al., 2015), a mechanism of action different from that of other bacteria. Further experiments from the same group showed that LexA binds to the predicted SOS operator sequence in the promoter region of *ssb1* (*alr0088*) and *ssb2* (*alr7559*), which are part of the SOS regulon (Kirti et al., 2017). This data supports the role of LexA as a repressor of the SOS genes in *Anabaena* PCC7120. However, different roles of LexA were also reported in cyanobacteria. Indeed, *Synechocystis* sp. PCC 6803, or *Gloeobacteor violaceus* PCC 7421 contains a non-cleavable LexA, suggesting a role other than that in the SOS reponse (Li et al., 2010). Further evidence showed that LexA from *Synechocystis* sp. PCC6803 regulates genes involved in carbon metabolism, phototactic motility and fatty acid synthesis (Kizawa et al., 2016, 2017), or those encoding RNA helicase and bidirectional hydrogenase (Domain et al., 2004). It is therefore possible that these unicellular cyanobacteria may lack a SOS response typical of that found in other bacteria.

In this study, we focused on *Anabaena* PCC 7120, in which the roles of DNA polymerases are so far rarely studied. It is not clear yet how the lack of DNA polymerases or interruption of DNA replication could affect other aspects of cellular processes, such as the cell cycle and heterocyst development. In a previous study, we created a conditional mutant of *polA* in *Anabaena* PCC 7120 by replacing its ribosome binding site (RBS) with a theophylline inducible riboswitch (TRS; Niu et al., 2018). Here, we constructed another conditional mutant of *dnaENI* via replacing its RBS with a synthetic CT promoter that is induced by copper and theophylline. Along with the previously constructed PolI mutant, we were able to demonstrate the essentiality of both DNA polymerase genes, *polA* and *dnaENI*, in *Anabaena* PCC 7120 and made a functional dissection of the DNA PolI and PolIII. Our results showed that except for the growth defect in both mutants, prevention of *polA* expression also resulted in morphological changes of vegetative cells and appearance of double heterocysts/proheterocysts possibly caused by cell division arrest, suggesting a complex relationship among chromosome replication, cell division, heterocyst development, and a possible SOS response. Our study showed how reduced expression of DNA polymerase genes exerts detrimental effects on cells of *Anabaena* PCC 7120 and opens a new window for our understanding on heterocyst development in cyanobacteria.

## Results

### Construction of Conditional Mutant of *dnaENI*

**FIGURE 1:**
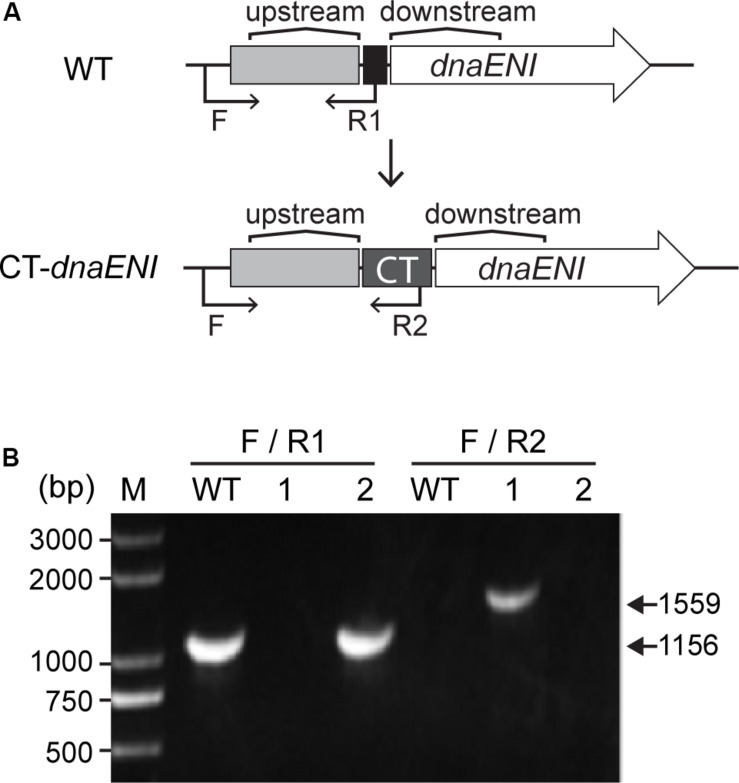
Construction of the conditional mutant CT-*dnaENI*. **(A)** The schematic representation of the genotype of the wild type (WT) and the CT-*dnaENI* strain. Upstream: upstream homologous arm, Downstream: downstream homologous arm. **(B)** Genotype verification of CT-*dnaENI* strain by PCR using the primers indicated as black arrows in **(A)**. WT was used as a control. F, R1, and R2 are short names for the oligonucleotides Pall3578F1165m, cr1_all3578F23mR, and Priboswitch2, respectively. The expected size of the PCR product amplified from the WT genome with F and R1 is indicated.

Since DNA replication is a crucial step in a cell cycle, disruption of genes involved in DNA replication could result in cell death due to loss of DNA. In *Anabaena* PCC 7120, *polA* (*alr1254*) gene is predicted to encode the PolI, and *dnaENI* (*all3578*) encodes the N-terminal portion of the split *dnaE* gene (Wei et al., 2006). In *E. coli*, DnaE is the alpha subunit of the PolIII holoenzyme and responsible for the bulk of DNA synthesis. Construction of *polA* and *dnaENI* gene knockout mutants in the traditional way by conjugation failed in *Anabaena* PCC 7120, however, we succeeded in knocking out the gene *alr2009* encoding the replication initiation factor DnaA, and the mutant did not affect cell growth nor heterocyst development, thus confirming a previous report showing that DnaA is not essential in *Anabaena* PCC 7120 (Ohbayashi et al., 2016; Supplementary Figure S1). Hence, we used a recently developed genetic tool based on CRISPR-Cpf1 (Niu et al., 2018), to create markerless replacement of the promoter region, or the ribosome binding region of essential genes. As described before, TRS-*polA* strain was constructed in a way that *polA* gene expression was under the control of a TRS, and the verification of its genotypes was previously reported (Niu et al., 2018). In this study, CT-*dnaENI* strain was constructed in a similar manner by replacing its native RBS with an artificial CT promoter (P*_*petE*_* promoter and theophylline riboswitch), which allowed gene expression in the presence of copper and theophylline (Figure 1A). Colonies were obtained after conjugation on plates under permissive conditions (containing both copper and theophylline). Subsequent PCR verification of the mutants with specific oligonucleotide primers showed that one out of the two tested colonies has the native RBS replaced by the CT sequence and the segregation was complete (Figure 1B). The obtained conditional mutant CT-*dnaENI* strain, together with previously constructed TRS-*polA*, were used for further characterization.

### Growth Phenotypes of *polA* and *dnaENI* Mutants in *Anabaena* PCC 7120

**FIGURE 2:**
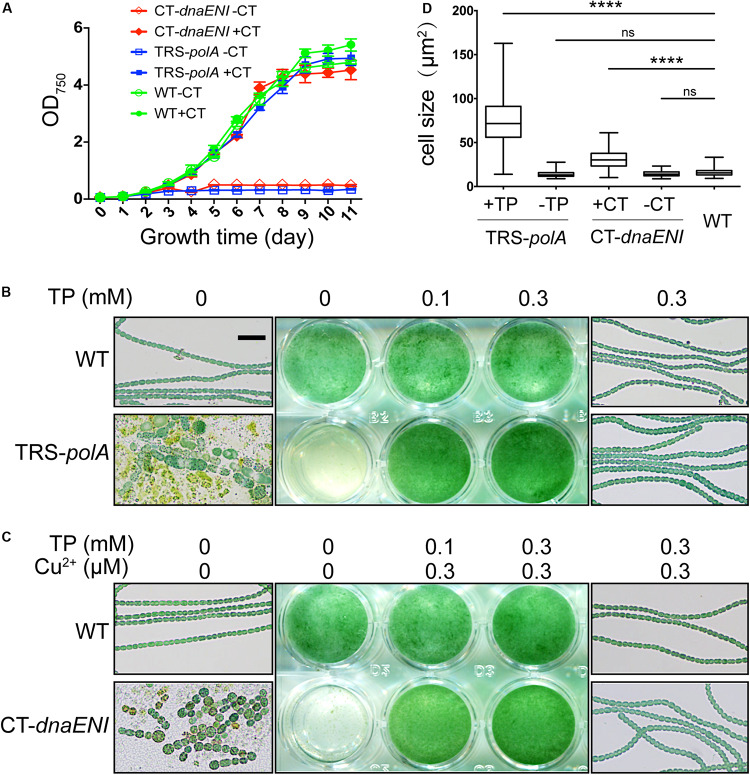
Growth of TRS-*polA* and CT-*dnaENI* under permissive and non-permissive conditions. **(A)** The growth curves of WT, TRS-*polA*, and CT-*dnaENI* in BG11 with or without CT (0.3 μM CuSO_4_ + 1 mM TP). **(B,C)** The growth of TRS-*polA* and CT-*dnaENI* cultured for seven days in BG11 with indicated levels of inducers. The middle-column images are cultures from the 24-well plates. On the left and the right column are representative microscopic images of cells from the wells with indicated levels of inducers. The scale bar of 20 μm is applied for all microscopic images and is shown in the upper-left image in **(B)**. **(D)** The cell size of WT, TRS-*polA*, and CT-*dnaENI* after 7-day’s cultivation in BG11 with or without inducers. 150 cells were measured for each strain and the mean cell sizes of TRS-*polA* and CT-*dnaENI* were significantly larger than that of WT according to a one-way ANOVA test (*P* < 0.05). ns: not significant. All experiments were done with three biological replicates.

Next we compared how mutations in genes encoding PolI or PolIII, respectively, affected cell viability in *Anabaena* PCC 7120. Under permissive conditions, namely in the presence of inducers, the growth of both TRS-*polA* and CT-*dnaENI* strains was similar to the wild type (WT). However, when shifted to non-permissive conditions by removing the inducers, cell growth for both mutants was arrested (Figure 2A). These results indicated that both genes were essential for cell growth in *Anabaena* PCC 7120. To further verify the growth phenotypes, we compared the cell growth under permissive and non-permissive conditions for both mutants with liquid cultures and microscopic examination. As shown in Figure 2B, a medium without added inducer did not support the growth of TRS-*polA* strain in BG11 medium as reported. Further microscopic examination revealed that cell viability was affected, with the majority of the cells lysed and the rest of them exhibiting irregular cell shape or enlarged cell size; some cells displayed a yellowish color indicating loss of pigmentation or in the course of collapsing (Figure 2B, lower left and Figure 2D). However, cultures with theophylline supplemented at a concentration of 0.1 mM or 0.3 mM restored cell growth without significant changes in cell morphology (Figure 2B, lower right and Figure 2D), consistent with the growth curves shown in Figure 2A. Similar experiment was also performed for CT-*dnaENI* strain by adding indicated levels of copper and theophylline in the growth medium (Figure 2C). As expected, the non-permissive conditions (lack of inducers) caused breakage of the filaments and the appearance of enlarged cells that eventually lysed within 7 days (Figure 2C, lower left), whereas cell growth was not affected when copper and theophylline were added in the cultures (Figure 2C, lower right and Figure 2D). These experiments demonstrated that *dnaENI* gene is essential for *Anabaena* PCC 7120 and further confirmed the essential function of *polA* as in a previous study (Niu et al., 2018).

### The Two Conditional Mutants Display Multiple Effects on Cell Cycle Events

**FIGURE 3:**
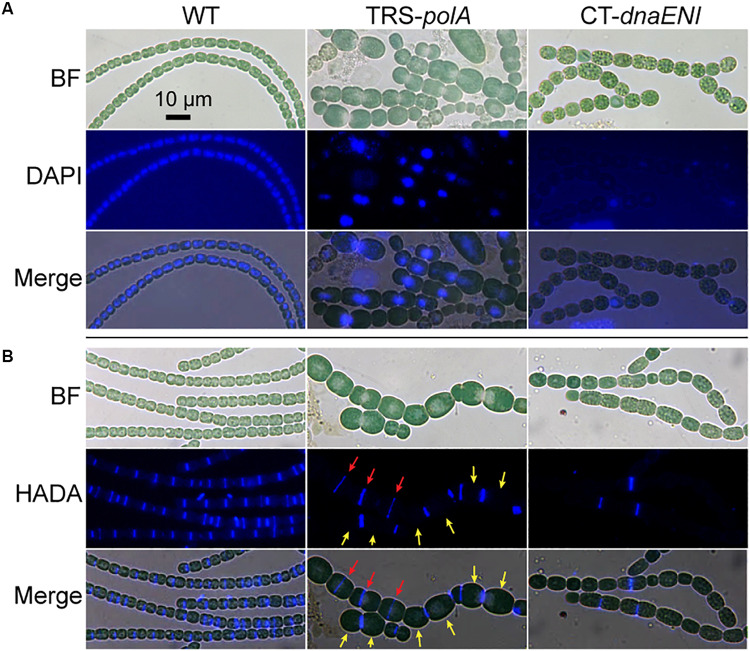
Cell morphology, DNA contents and cell division in the conditional mutants. **(A)** Visualization of the nucleoids in WT, TRS-*polA*, and CT-*dnaENI* with DAPI staining. **(B)** Septum formation indicated by HADA labeling in WT, TRS-*polA*, and CT-*dnaENI*. In both **(A)** and **(B)**, cells were cultured under non-permissive conditions for 7 days before sampled for imaging. All experiments were performed at least three times. BF: bright field. Scale bar of 10 μm is applied to all images.

To gain more information on how depletion of DNA polymerases led to cell death, we performed DAPI staining to visualize DNA contents in the mutant cells. As a control, after DAPI staining, and WT cells displayed evenly distributed nucleoid occupying a large portion of the cells (Figure 3A, left panel). After growth under non-permissive condition for 7 days, TRS-*polA* cells exhibit a severe problem in chromosome segregation. Indeed, DAPI fluorescence indicated that some cells have no DNA, while in many others, the chromosomes were locked at the junction between the two daughter cells, thus unable to complete the segregation. These observations indicated the cell cycle cannot be completed due to a failure in chromosome segregation (Figure 3A, middle panel). For CT-*dnaENI* strain, no DAPI fluorescence was detected in most of the cells after being cultured under non-permissive conditions for 7 days, indicating that a lack of a functional PolIII led to DNA-less cells, likely because DNA polymerase III acts as the major enzyme for DNA elongation (Figure 3A, right panel). For both mutants, increasing cell lysis was observed over time (Figure 3).

For both mutants, changes in cell shape were observed, and this was particularly obvious for TRS-*polA*, since most of the cells of this strain were enlarged and longer as compared to the WT cells, with occasional asymmetric-cell division events being found (Figure 3). These results indicated that cell growth and division were abnormal, suggesting that cell wall synthesis, or cell division may be affected in these mutants. A major component of the cell wall, peptidoglycan (PG), is important for maintaining the bacterial cell shape. Although rigid, PG layer is yet dynamic so it can be constantly remodeled to pace up with cell growth and division, in particular at the division site where the naissance of the new cell poles requires destruction and reconstruction of the PG layer during cell constriction (Egan and Vollmer, 2013). 7-Hydroxycoumarin-amino-D-alanine (HADA) is a fluorescence analog of the PG synthesis precursor D-Ala and has been previously proven useful for the labeling of active PG synthesis sites at the septa in different cyanobacteria, serving as a good marker for the later steps of cell division once initiated by FtsZ (Zhang et al., 2018). In cells of the WT, HADA fluorescent signal was mainly found at the septal areas (Figure 3A, left panel), as well as the mid-cell positions for elongated cells where physical constriction was not yet visible, as reported previously (Zhang et al., 2018). Except for a few cells (Figure 3B red arrows), no septum formation (indicated by HADA signal) is detected at the mid-cell position for most of the large cells in the TRS-*polA* strain (Figure 3B, yellow arrows), which normally exhibit active cell division as in the wild type. These results suggested that PG synthesis at the septal sites was blocked under such conditions. Under the non-permissive conditions, CT-*dnaENI* strain exhibited similar results as the TRS-*polA* strain, with remnant fluorescence seen only in a few cells (Figure 3B, right panel). Altogether, these results showed that when DNA replication was disrupted, the cells displayed a loss of PG remodeling activity at the division sites, thus blocking septum formation.

**FIGURE 4:**
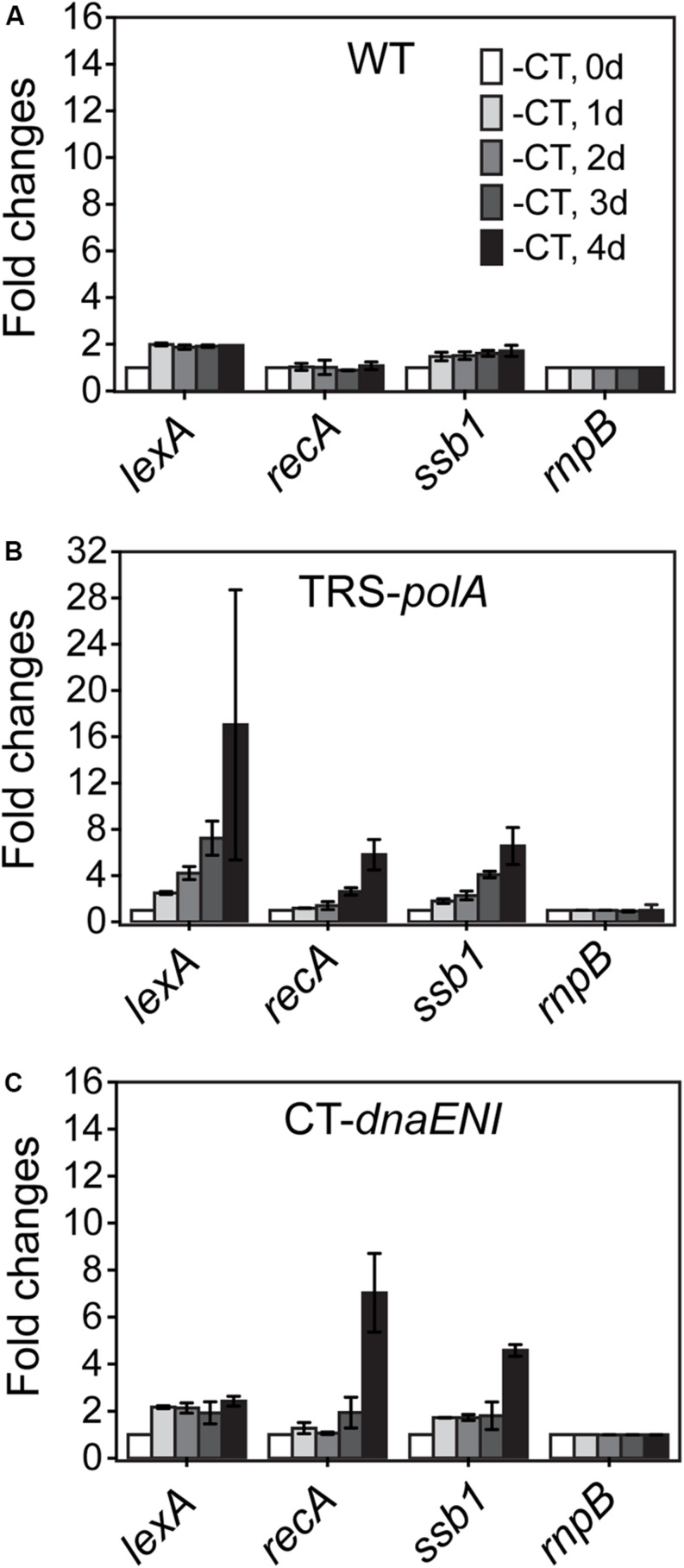
The transcription levels of *lexA*, *recA*, *ssb* genes in WT **(A)**, TRS-*polA* **(B)**, and CT-*dnaENI* **(C)** quantified from RT-qPCR assays. -CT: without added CuSO_4_ and TP in the growth medium. The transcription levels of genes were tested at the beginning of non-permissive growth (0 day), then everyday up to 4 days following transfer to non-permissive conditions, as indicated 1 day, 2 day, 3 day, and 4 day in **(A)**. The experiment was done with three technical replicates in two biological replicates.

The observed phenotype in cell division for both mutants could be resulted from a defect in chromosome segregation whose failure could affect cell division; alternatively, DNA segregation and cell division were coordinated through a global control system such as the SOS response mechanism. In bacteria, the SOS response is a general mechanism induced upon DNA damage and plays a role in coordinating different cell cycle events in order to prevent the damaged DNA being passed to the daughter cells. We would like to know whether similar mechanism exist in both conditional mutants. To check the induction of a possible SOS-like response, transcription levels of three key SOS gene homologs, *lexA*, *recA*, and *ssb1*, were measured over time by quantitative Real-Time PCR (qRT-PCR). While the transcription levels remained relatively stable for all tested SOS homologous genes in the WT cells (Figure 4A), culturing of the TRS-*polA* strain under non-permissive conditions led to gradually increased expression of all the three genes examined over time, especially for *lexA*, whose level jumped over 16-fold at day four (Figure 4B). Induction of SOS homologous genes in the CT-*dnaENI* strain under non-permissive conditions showed a different pattern, with a delayed increase in the expression of *recA* and *ssb1* at day four, but no significant changes for the expression of *lexA* was observed (Figure 4C). Our previous experiments (Figure 3) have showed that severe DNA loss was starting to occur after four-day growth and little DNA was revealed by DAPI staining at day seven in the CT-*dnaENI* strain with extensive cell lysis, so the samples were taken only up to day four in this experiment. Thus, a stronger induction of SOS homologous genes was observed in TRS-*polA* as compared to CT-*dnaENI*, although with different expression patterns for the three genes studied.

### Conditional Mutants Displayed Abnormal Heterocyst Developmental Process

**FIGURE 5:**
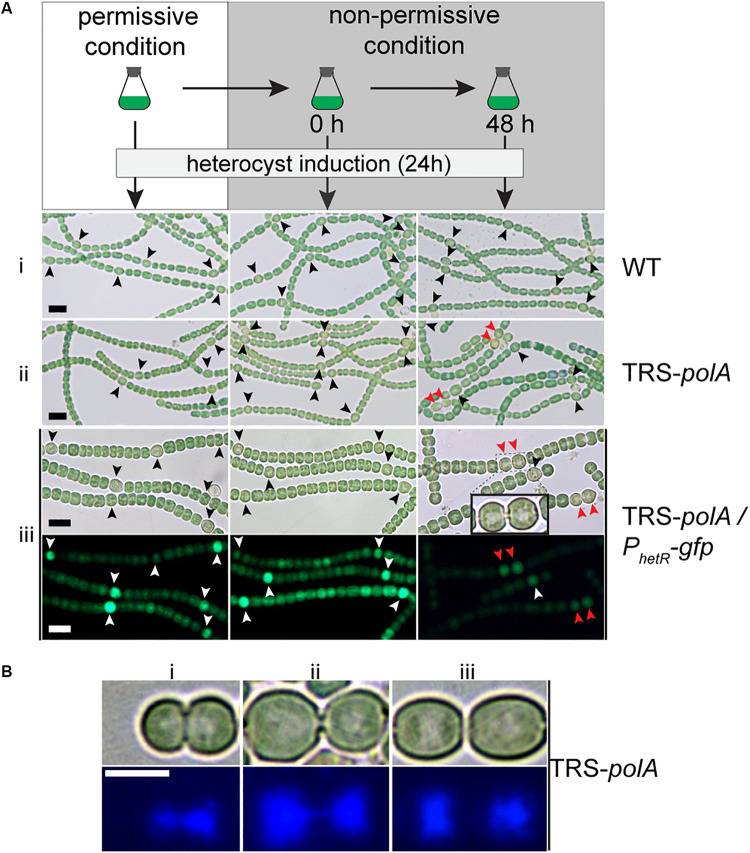
Heterocyst formation in DNA polymerases mutants under non-permissive conditions. As illustrated in **(A)**, all strains were initially cultivated in BG11 with inducers to allow gene expression (permissive condition). Heterocyst induction was performed at 0 h and 48 h after the inducers were removed from the growth medium (non-permissive condition). After 24 h of induction, images of heterocyst formation under a light microscope were shown for WT (i) and TRS-*polA* (ii). In order to monitor heterocyst development in the mutants, transcriptional fusion of *hetR* promoter with *gfp* (P*_*hetR*_*-*gfp*) was introduced into TRS-*polA* (iii) and the formation of heterocysts was recorded in both Brightfield (upper panel) and fluorescent channels (lower panel). Black or white arrows indicate single heterocyst and the red arrows indicate double heterocysts/proheterocysts. Scale bars: 10 μm. **(B)** DAPI staining of double heterocysts/proheterocysts in TRS-*polA* strain grown under non-permissive conditions. Bright field images (upper panel) and fluorescent images (bottom panel) showed presence of double heterocysts/proheterocysts at three different stages of nucleoid segregation (i, ii, and iii). Experiments were performed twice. Scale bar: 5 μm.

Interaction between the process of heterocyst differentiation and cell cycle has been suggested in previous reports (Sakr et al., 2006a). With the conditional mutants in hand, it became possible to check if DNA replication process were involved in the regulation of heterocyst differentiation. Precultures of the two mutants together with the WT as a control, were first prepared in BG11 containing nitrate as a combined nitrogen source, under permissive conditions (Figure 5). The cultures were then shifted to non-permissive conditions to arrest the expression of either *polA* (TRS-*polA*) or *dnaENI* (CT-*dnaENI*), followed by induction of heterocyst differentiation in BG11_0_ at different time points (0 h or 48 h) after the change from permissive to non-permissive conditions. An incubation period longer than 48 h for both mutants under non-permissive conditions started to affect the cell viability, thus was no longer suitable for testing the effect on heterocyst differentiation. In all experiments, images were captured 24 h after the induction of heterocyst in BG11_0_ medium. As shown in Figure 5, heterocyst formation was normal in both conditional mutants at time zero upon removal of inducers, comparable to the WT or the corresponding strains grown under permissive conditions (Figure 5A i,ii, left and middle column). When heterocyst differentiation was induced 48 h after the change toward non-permissive conditions, TRS-*polA* showed development of double heterocysts/proheterocysts (accounting for 25% of all heterocysts), while CT-*dnaENI* strain formed single heterocysts as the WT (Supplementary Figure S2). Note that an increase of heterocyst frequency (represented by reduced number of interval cells, Supplementary Figure S3) was observed in both conditional mutants at 48 h, from 7.3% in the WT to 10.7% and 10.9% in the TRS-*polA* and CT-*dnaENI*, respectively.

So far, contiguous heterocysts, known as the Mch (multiple contiguous heterocysts), have been reported in several mutants, such as *hetR*-overexpressing strain, or *patS* mutant (Buikema and Haselkorn, 1991; Yoon and Golden, 1998; Risser and Callahan, 2008). However, in the case of the TRS-*polA* strain, only double heterocysts/proheterocysts, instead of strings of contiguous heterocysts, were observed. In order to understand this phenotype, we introduced into the TRS-*polA* strain a transcriptional fusion expressing GFP driven by the promoter of *hetR* (P*_*hetR*_*-*gfp*) which could be used as a molecular marker to follow heterocyst development before the occurrence of morphological differentiation (Black et al., 1993). In the presence of inducers (under permissive conditions), or at time zero upon removal of the inducers (non-permissive conditions), single heterocysts with a high GFP fluorescence were observed, similar as the WT control (Figure 5A iii, left and middle column). When heterocysts were induced 48 h after the transfer to non-permissive conditions, double heterocysts/proheterocysts that were both highly fluorescent were found in TRS-*polA* but not the WT (Figure 5C, right column). Pairs of proheterocysts could be observed after 12 h of nitrogen step-down (data not shown). Longer growth period in the absence of inducers led to cell death in TRS-*polA*, which made detection of heterocyst development unreliable and therefore excluded in this experiment. Taken together, these results indicated that disruption of DNA replication due to lack of PolI/PolIII may affect the heterocyst pattern formation.

Many double heterocysts/proheterocysts of TRS-*polA* displayed a long, narrow neck connecting the two heterocyct cells (Figure 5A iii inlay). Within each heterocyst, an area that appeared light-pale could be observed under a light microscope. According to our observation shown in Figure 3, we speculated that this area may correspond to nucleoids and could be stained with DAPI. Interestingly, such materials were also found inside the neck connecting the two heterocysts, suggesting that the two nucleoids were still tangled together and unable to segregate into the two daughter cells, reminiscent of the results seen in Figure 3. To test our hypothesis, we performed DAPI staining with TRS-*polA* cells grown under non-permissive conditions as described previously. Indeed, the light-pale region corresponds well to the space occupied by nucleoids indicated by the fluorescent signal. Moreover, three different stages of double heterocysts/proheterocysts were found (Figure 5B), with many of them exhibited either a thick or a thin neck region (Figure 5B i,ii). DAPI staining of the same cells showed the nucleoids of most double heterocysts/proheterocysts cells are not separated, either largely tangled together (Figure 5B i), or linked via a string of DNA (Figure 5B ii). Occasionally, double heterocysts/proheterocysts with complete segregation of the nucleoids were also detected (Figure 5B iii). These results indicated that the observed double heterocysts/proheterocysts were descended from the same parental cell that has difficulty in completely segregating the two daughter nucleoids. As a consequence, the two daughter cells were unable to complete cell division on time, causing a prolonged period during which the two daughter cells were connected with an elongated neck filled with DNA.

## Discussion

Understanding the function of essential genes is important for us to learn the survival mechanism of an organism. A few genetic tools for creating conditional mutant of essential genes have been developed in *Anabaena*, such as the NtcA-dependent promoter P_ND_ and the Co^2+^/Zn^2+^ inducible promoter coaT (Ramos-León et al., 2015; González et al., 2016; Wegelius et al., 2018). Such types of promoters allow conditional expression or shutdown of target proteins depending on the presence of specific inducers, and proved to be useful and effective for studying essential genes. Previously we have reported creation of a conditional mutant of DNA polymerase gene (*polA*) by replacing its RBS with a synthetic TRS, a technique that was validated by showing the essential function of *polA* (Niu et al., 2018). In the present study, we have tried making another mutant of *dnaENI* with the same technique but only succeeded when using a different inducible synthetic CT promoter. We had no adequate explanation for this difference, although the CT promoter may allow a more stringent regulation of gene expression through the dual control of copper and theophylline. Based on our experiences, we advise trying both synthetic promoters for engineering of conditional mutants in this organism.

The functions of DNA polymerases in cyanobacteria are rarely studied as compared to that in *E. coli*, in which PolIII and PolI are the two polymerases responsible for DNA replication. PolIII is a large complex and the main enzyme polymerizing nucleotides on both leading and lagging strands, whereas PolI plays an essential role in maturation of Okazaki fragments by removing RNA primers (by its 5′-3′ exonuclease activity) and filling the gaps (by its DNA polymerase activity), as well as repairing damaged DNA (Larry et al., 2013). Here, by relying on the conditional mutants, we were able to confirm that both *polA* and *dnaENI* genes were essential in *Anabaena* PCC 7120, because in both cases, switching from permissive to non-permissive growth condition induced cell death. These results contrast with the fact that DnaA is not essential for the initiation of DNA replication (Ohbayashi et al., 2016), which has been also confirmed in our result (Supplementary Figure S1). Notably, cells lacking PolI are still able to grow in *B. subtilis* and *E. coli*, unless synthetic mutation with *ypcP* (for *B. subtilis*) or *xni* (for *E. coli*) encoding a protein with a 5′-3′ exonuclease domain was introduced, indicating a redundant function of PolI and a second exonuclease in these organisms (Fukushima et al., 2007). Sequence similarity search in *Anabaena* PCC 7120 using the 5′-3′ exonuclease domain sequence of YpcP or Xni returned no hit except PolI alone, suggesting that PolI is the sole protein having a 5′-3′ exonuclease domain in the genome of *Anabaena* PCC 7120. This provided a rational for the essential function of *polA* in this cyanobacterium (Fukushima et al., 2007).

Despite the fact that both proteins are essential, lack of PolI or PolIII exhibits some common as well as different phenotypes in *Anabaena* PCC 7120. For both conditional mutants under non-permissive conditions, they displayed multiple phenotypes including changes in cell morphology, in cell division and also in heterocysts frequency (Figure 3 and Supplementary Figure S2). Increases in cell size were observed in both mutants and likely caused by the cell division defect due to disruption of DNA replication. In most of the cells of both mutants, no PG remodeling activity was detected with HADA labeling (Figure 3), suggesting that cell constriction could no longer take place after a prolonged arrest of DNA replication. We therefore could not exclude the possibility that the increased heterocyst frequency observed in both mutants were due to the lack of cell division in vegetative cells. Note that in the CT-*dnaENI* mutant, cell elongation was observed only after a long-time blockadge of DNA replication, at a time when most of cells contained no detectable DNA (Figure 3). This observation suggested that the event of cell division could still occur after switching to non-permissive conditions, but ceased when the intracellular DNA/proteins levels dropped substantially due to lack of DNA replication. However, it remains unclear when cell division started to be affected once *dnaENI* expression was blocked, so we could not determine if it was the lack of DNA replication or that of genetic material itself, that caused the phenotype related to cell division in the mutant. In the case of the *polA* mutant, the phenotype on cell division is more likely caused by the disorder in chromosome segregation (Figure 3). Cells of TRS-*polA* strain often displayed unfinished DNA segregation, with two nucleoids still connected at the cell-cell junctions. Such a phenotype was not observed for the CT-*dnaENI* strain and this difference may be explained by the different roles of the two polymerases in DNA synthesis. Different from that of CT-*dnaENI* mutant, no nucleoid-less cells were found in the TRS-*polA* strain when cells were grown under non-permissive conditions for 7 days (Figure 3). This observation makes sense if we consider the different roles of the two DNA polymerases. PolI plays a role in the maturation of Okazaki fragments and DNA repair, while PolIII synthesizes the bulk of DNA for both leading and lagging strands in the cells. Therefore, the overall DNA content should be less affected in the TRS-*polA* strain, but should decrease over time in the CT-*dnaENI* mutant, leading to cells that had no DNA or little DNA that could not be detected by DAPI staining. These results indicated the complexity of cell cycle in *Anabaena* and further studies will be necessary to investigate the underlying mechanism.

The two conditional mutants had a difference in the expression of genes predicted to be involved in a SOS response, which is a general mechanism for coordinating the cell cycle in order to prevent damaged DNA from being inherited by the daughter cells (Maslowska et al., 2019). Previous studies suggested that a SOS-like response may also exist in *Anabaena*, but the mechanism may differ as compared with *E. coli*. The three genes *lexA*, *recA*, and *ssb1* are among the genes most extensively studied in *E. coli* and much less in *Anabaena* (Kirti et al., 2013, 2017; Kumar et al., 2015). *ssb1* was reported to be under the control of LexA and its expression is enhanced following exposure to DNA damaging factors such as mitomycin C (Kirti et al., 2013, 2017). Therefore, we chose these three markers in this study. As expected, induction of SOS genes did occur in both conditional mutants, but more significantly in the TRS-*polA* strain. This difference may be caused by the different damaging effect of PolI or PolIII deficiency in the cells. According to the role of DNA polymerases in bacteria, the lack of PolI is expected to cause accumulation of Okazaki fragments and exposure of single stranded DNA due to unfilled gaps as well as a deficiency in DNA repair. On the other hand, the lack of PolIII caused DNA-less cells, thus unable to sustain gene expression. The strong induction of *lexA* in the TRS-*polA* strain, together with the arrest of cell division, could allow DNA repair to occur. However, the Okazaki fragments are about 2-kb long in average, hence, the large number of gaps on the chromosome and the lack of PolI itself make such repair futile, thus leading to cell death in the end. Overall, we observed a phenomenon reminiscent of the SOS response, but more studies will be needed to further define the nature of this response and its relationship with DNA replication.

Heterocyst formation still occurred in both mutants and PolI depletion caused a high frequency of double heterocysts or proheterocyst. Double heterocysts/proheterocyts have been previously reported, such as in *patS*, *hetN*, or *patU3* knockout strains and *hetR* overexpression strain (Buikema and Haselkorn, 1991; Borthakur et al., 2005; Zhang et al., 2007; Risser and Callahan, 2008; Corrales-Guerrero et al., 2014). When an extra copy of *hetR* carried on a replicative plasmid was present in the cells of *Anabaena* PCC 7120, pairs of heterocysts were observed and described as being dividing or unseparated, indicating the dividing cells could respond to nitrogen deprivation and proceed with differentiation (Buikema and Haselkorn, 1991). In addition, *patS*, *hetN*, *patU3* knockout, and overexpression of *hetR* also caused Mch, something that we did not observe in the TRS-*polA* strain. Our studies (Figure 5) pointed out how these double heterocysts/proheterocysts could have occurred. In the WT, a neighboring cell may all tend to respond to nitrogen starvation, but lateral competition involving inhibitory signals such as diffusible PatS peptides, resolve them so that only a single heterocyst will become a mature one (Yoon and Golden, 1998; Flores et al., 2019). In the case of the TRS-*polA* strain, the presence of unsegregated DNA in the neck region between the two daughter cells prevented or significantly slowed down the completion of cell division (Figure 5B). Non-segregated nucleoids thus resulted in delayed closure of a septum, which allows free diffusion of signaling molecules in the two daughter cells. In such a case, the failure of competition between the two cells through the cell-autonomous activator HetR and the inhibitory signals such as PatS peptides acting in a non-cell autonomous manner, would lead to both cells to become heterocysts. The phenotypes of the TRS-*polA* strain is reminiscent of those observed for inactivation of the *ftsK* gene, which is involved in the coordination of cell division with the completion of chromosome segregation at the division site (Dewachter et al., 2018). The fact that double heterocysts/proheterocysts, whose differentiation was initiated during cell division, only consist of about 25% of all the heterocyst observed is a genetic evidence suggesting that cells at different cell cycle stages could all respond to nitrogen starvation. This observation is also consistent with the result obtained by cell lineage analysis (Asai et al., 2009).

By relying on the conditional mutants, we could confirm the essential function of both PolI and DnaENI in *Anabaena* PCC 7120. Furthermore, our studies indicate the coordination of DNA replication with cell division and chromosome segregation, as well as between chromosome segregation and cell differentiation. However, we do not know how much of such coordination could be attributed to the SOS-like response mechanism, or other mechanisms that remained to be uncovered. Nevertheless, the present study provides useful genetic materials for the understanding of the roles of DNA polymerases in cyanobacteria.

## Materials and Methods

### Strains and Growth Conditions

All strains used in this study were listed and described in Supplementary Table S1. *Anabaena* PCC 7120 strains were grown in BG11 (Stanier et al., 1971) or in BG11_0_ medium (BG11 without combined nitrogen). All strains were cultivated at 30°C in a shaker with the speed of 180 rpm and the light intensity of 30 μmol photons m^–2^ s^–1^. To induce gene expression, 0.3 μmol CuSO_4_ and 1 mM TP (theophylline) were used for CT-*dnaENI* strain and 1 mM theophylline was used for TRS-*polA* strain (Niu et al., 2018). Whenever necessary, 5 μg mL^–1^ spectinomycin and 2.5 μg mL^–1^ streptomycin, or 100 μg mL^–1^ neomycin were added to the medium.

### Construction of Plasmids and Mutant Strains of *Anabaena* PCC 7120

All plasmids were verified by Sanger sequencing and listed in Supplementary Table S2. Primers used in this study were listed in Supplementary Table S3.

Plasmid pCpf1-CT- *dnaENI* is constructed for making conditional mutant CT-*dnaENI*. To generate the repair template, a region upstream of *dnaENI* amplified using the primers Pall3578F940m and Pall3578R150m, a region of CT promoter (an artificial promoter with a *petE* promoter and a theophylline riboswitch) amplified using the primers Pall0258F475m and PV_19 from vector pCT, and a region downstream amplified using the primers Pall3578F1 and Pall3578R960 were fused by overlapping PCR using the primers Pall3578F940m and Pall3578R960. A sequence of two spacers was prepared by annealing the complementary primers cr1_all3578F23mF, cr1_all3578F23mR, cr2_all3578R32mF, and cr2_all3578R32mR. The repair template and the spacer sequence were sequentially cloned into pCpf1 at the sites of *Bgl*II/*Bam*HI and *Aar*I/*Aar*I, resulting in the mutation plasmid pCpf1-CT-*dnaENI*.

To generate plasmid pCint2-Δ*dnaA*, the upstream and downstream sequences of dnaA gene as well as the kanamycin resistance cassette were amplified using specific primers listed in Supplementary Table S2. The resulting three fragments were fused by overlapping PCR using the primers Palr2009F1255m and Palr2009R2628. This repair template was then cloned into pCint2 at the sites of *Bgl*II/*Bam*HI, resulting in the plasmid pCint2-Δ*dnaA*.

To construct the CT-*dnaENI* conditional mutant, the mutation plasmid pCpf1-CT-*dnaENI* was transferred into *Anabaena* PCC 7120 by conjugation (Cai and Wolk, 1990; Elhai et al., 1997). The exconjugants were selected on BG11 plates containing 100 μg mL^–1^ neomycin, 0.3 μmol CuSO_4_, and 1 mM theophylline and verified by PCR and Sanger sequencing.

To construct WT/pP*_*hetR*_*-*gfp*, TRS-*polA*/pP*_*hetR*_*-*gfp*, and CT-*dnaENT*/pP*_*hetR*_*-*gfp*, the replicated plasmid pP*_*hetR*_*-gfp was transferred into WT, TRS-*polA*, and CT-*dnaENT*, respectively, by conjugation. The positive clones were selected on BG11 plates containing 5 μg mL^–1^ spectinomycin and 2.5 μg mL^–1^ streptomycin and verified by PCR.

To construct Δ*dnaA*, the mutation plasmid pCint2-Δ*dnaA* was transferred into *Anabaena* PCC 7120 by conjugation, and the single-crossover was selected on BG11 plates containing 100 μg mL^–1^ neomycin and verified by PCR. The homologous double-crossover was selected on BG11 plates containing 8% sucrose, verified by PCR and Sanger sequencing.

To make the plasmid for transcriptional fusion of *hetR*, the promoter region of *hetR* was amplified using the primers Palr2339F915m and Palr2339R3 and subsequently cloned into pRL25N-Lgfp at the sites of *Bam*HI/*Xho*I, resulting in the transcriptional fusion plasmid pP*_*hetR*_*-*gfp*. It was then moved into the cells by conjugation.

### Viability Test for TRS-*polA* and CT-*dnaENI* Cells

All strains were cultivated to the log phase and then washed with BG11 for three times to remove inducers. Cells were resuspended in fresh BG11 containing gradient inducers to OD_750_ of 0.08 in a 24-well plate and cultured at the same conditions for 4 days. The cultures were diluted once more into another 24-well plate to OD_750_ of 0.08 with the same induction and continued culturing for another 3 days. WT *Anabaena* PCC 7120 was used as a control. Finally, observed the phenotype and took pictures. The concentrations of inducers (theophylline for TRS-*polA* and theophylline in combination with CuSO_4_ for CT-*dnaENI*) were used as indicated in Figure 2.

### HADA Labeling, DAPI Staining and Heterocyst Induction

HADA was synthesized, used and stocked as previously described (Kuru et al., 2015; Zhang et al., 2018). All strains were cultivated to the log phase, washed with BG11 for three times to remove inducers before resuspended in 100 ml of fresh BG11 to OD_750_ of 0.08. The cultures were grown for 4 days and then diluted once more to OD_750_ of 0.08 and cultivated for another 3 days. For HADA labeling, cells were taken at day 6, washed twice with BG11 and resuspended in fresh BG11 containing 200 μM HADA to OD_750_ of 0.2–0.3. After 24 h, cells were washed three times with BG11 to remove the residual HADA before subjected to microscopy observation. For DAPI labeling, samples were prepared at day 7 by adding DAPI to the final concentration of 2 μg/ml and placed in dark for 20 min. Then the samples were used for microscopy observation. To exam heterocyst formation, samples prepared at day 0, day 2, day 4, and day 6, respectively, washed twice with BG110 and resuspended in BG110 to OD_750_ of 0.2–0.3. After 24 h’s nitrogen step-down, samples were prepared, and observed for phenotypes.

For microscopic studies, a SDPTOP EX30 microscope was used to acquire brightfield images and a SDPTOP EX40 for fluorescent images. HADA has maximal excitation wavelength of 405 nm and maximal emission of 460 nm. Exposure time of 200 ms was used for acquiring both HADA and DAPI fluorescent images. All images were analyzed using ImageJ.

### Analysis of Cell Size, Heterocyst Frequency and Double Heterocysts/Proheterocysts

For cell size, images taken under a microscope after 7 days without inducer were used for analyzing the cell size. The length and the width of 150 cells were measured, respectively, using ImageJ and the size of each cell was estimated by multiplying its length and width. For TRS-*polA*, the width of an irregular cell was measured at the widest position.

Microscopic images taken after 3-days’ cultivation without inducer and 1-day with nitrogen step-down were used for analyzing the vegetative cell intervals and the frequency of double heterocyst/proheterocysts. For each strain, the vegetative cell intervals of more than 200 pairs of heterocyst were analyzed. For double heterocyst/proheterocysts frequency analysis in TRS-*polA*, more than 110 heterocysts were analyzed with three parallel analysis.

### Quantitative Real-Time PCR

Wide-type *Anabaena* PCC 7120, TRS-*polA*, and CT-*dnaENI* were cultivated to the log phase in BG11 with 0.3 μmol CuSO_4_ and 1 mM theophylline and then washed with BG11 for three times to remove the inducers. Cells were resuspended in 150-ml fresh BG11 to OD_750_ of 0.3 and cultured at the same conditions for 4 days. To prepare the samples, cultures were diluted to the initial concentration in fresh BG11 and 50 ml of samples were harvested at day 0, day 1, day 2, day 3, and day 4, respectively, with two parallel experiments. Total RNA was extracted with Plant Total RNA Isolation Kit (FOREGENE) and reverse transcribed with HiScript Q RT super mix (Vazyme). qRT-PCR was performed using ChamQ SYBR Qpcr Master Mix (Vazyme, Nanjing, China) with specific primers listed in Supplementary Table S2.

## Data Availability Statement

The raw data supporting the conclusions of this article will be made available by the authors, without undue reservation, to any qualified researcher.

## Author Contributions

C-CZ and XZ designed the study. W-YX and LX performed the experiments. C-CZ, XZ, W-YX, and YY analyzed the data. C-CZ, W-YX, and YY wrote the manuscript.

## Conflict of Interest

The authors declare that the research was conducted in the absence of any commercial or financial relationships that could be construed as a potential conflict of interest.
