# Supplementary material for: Functional Dissection of Genes Encoding DNA Polymerases Based on Conditional Mutants in the Heterocyst-Forming Cyanobacterium Anabaena PCC 7120
Source: Front Microbiol. 2020 Jun 3;11:1108. doi: 10.3389/fmicb.2020.01108 (PMC7283527; doi:10.3389/fmicb.2020.01108)
Supplement: Supplementary file 1 [file Data_Sheet_1.docx]

Supplementary Material

# Supplementary Tables

**Table S1. All strains used in this study.**

| **Strains** | **Descriptions** | **Sources** |
| --- | --- | --- |
| *Anabaena* PCC 7120 | Wild type | Pasteur Culture Collection |
| TRS-*polA* | Conditional mutant of *polA* (*alr1254*), the theophylline riboswitch replaces the native riboswitch of *polA* | (Niu et al., 2018) |
| CT-*dnaENI* | Conditional mutant of *dnaENI* (*alr3578*), CT promoter replaces native promoter of *dnaENI* | This study |
| Δ*dnaA* | *dnaA* markless deletion; Nm^r^ | This study |

**Table S2. All plasmids used in this study.**

| **Plasmids** | **Descriptions** | **Sources** |
| --- | --- | --- |
| pCT | Km^r^Nm^r^; | Accession number: MK948095 |
| pCpf1 | Km^r^ Nm^r^; CRISPR-Cpf1-Based Genome Editing vector | (Niu et al., 2018) |
| pSfgfp-Sp | Sm^r^ Sp^r^; carrying supper folding *gfp* coding sequence | Accession number: MK948098 |
| pRL25N-Lgfp | Km^r^ Nm^r^; carrying *gfp* coding sequence | (Zhang et al., 2013) |
| pCint2 | Km^r^Nm^r^; *sacB*-bearing cloning vector | This study |
| pCpf1-CT-*dnaENI* | Km^r^Nm^r^; *sacB*-bearing cloning vector | This study |
| pCint2-*ΔdnaA* | Km^r^ Nm^r^; for *dnaA* markless deletion | This study |
| pP*_hetR_*-*gfp* | Km^r^ Nm^r^; pRL25N-Lgfp carrying P*_hetR_*-gfp fusion | This study |

| **Name** | **Sequences (5’ - 3’)** |
| --- | --- |
| Pall3578F940m | GCAGAAATTCGATATCTAGATTGAATTACGGAATGCAACCGTA |
| Pall3578R150m | CAGGCTTTAGGATCCAAGATATCTTATGTCCCCAGTCC |
| Pall0258F475m | CTTGGATCCTAAAGCCTGTGAAATTAACTG |
| PV_19 | CATCTTGTTGTTACCTCCTTAGCA |
| Pall3578F1 | TGCTAAGGAGGTAACAACAAGATGTCCTTTGTACCTTTACATATTC |
| Pall3578R960 | CGCAACGTTGTTGCCATTGCGGATTTACGATTGAGTTTCTCCAA |
| cr1_all3578F23mF | AGATTGGGCTAGCGCTGGGAGAGAGT |
| cr1_all3578F23mR | ATCTACAACAGTAGAAATTACTCTCTCCCAGCGCTAGCCCA |
| cr2_all3578R32mF | AATTTCTACTGTTGTAGATCCCTGGGATTTCTTGTGAGCCA |
| cr2_all3578R32mR | AGACTGGCTCACAAGAAATCCCAGGG |
| Pall3578F1165m | GCCTGAGCAGAGATTGGTACTA |
| Priboswitch2 | tcttgttgttacctccttagcagggtgctgccaagggcatcaagacgatgct |
| Palr2009F1305m | ATATTGATGCGATCGCCAAA |
| Palr2009F1255m | GCAGAAATTCGATATCTAGATCCGTGAGGGTTGCCATCTG |
| Palr2009R1ma | cgcaacgttgttgccattgcGTGCTTGAATATCTGCAATCAA |
| Palr2009R850 | cgcaacgttgttgccattgcGTGCTTGAATATCTGCAATCAA |
| Palr2009F1378a | ACCGGATCATCAGTACTCCCTGATCATAAAAATTCGACGTTTTC |
| Palr2009R2628 | CGCAACGTTGTTGCCATTGTCGGCGTTCAATGGTCAC |
| Pinsert6 | gggagtactgatgatccggtAGTTATTGCTTACTTTTCGAATTGTG |
| PtstspF | AGCACTAGCGTCGGTAGCGCT |
| Pgfp_spR | GGGAGTACTGATGATCCGGT |
| Palr2339F915m | GCTTGGGCTTGCGGCCGCATGTTTGGTCACTTAGGTAGCAGGA |
| Palr2339R3m | GCCCTTGCTCACCATATGACAAATAGTTGAATAGCACGCTTA |
| Palr4908F1 | ATGGAACGCCTAACAGAA |
| Palr4908R75 | CGAATGCTGGTGAATACG |
| Pall3272F882 | CAGTTACAACGGTGATAA |
| Pall3272R1071 | TTCTTCCTCTTCTACATCT |
| Palr0088F87 | ATTAACTCTAGCCGTCAATC |
| Palr0088R170 | ACCTCTGCTGTCTTATCC |
| Pallrs04F334 | CCAGTTCCGCTATCAGAGAG |
| Pallrs04R456 | GAGGAGAGAGTTGGTGGTAAG |

**Table S3. Primers used in this study.**

**References**

Niu, T.-C., Lin, G.-M., Xie, L.-R., Wang, Z.-Q., Xing, W.-Y., Zhang, J.-Y., et al. (2018). Expanding the potential of CRISPR-Cpf1 based genome editing technology in the cyanobacterium Anabaena PCC 7120. *ACS Synth. Biol.* doi:10.1021/acssynbio.8b00437.

Zhang, S.-R., Lin, G.-M., Chen, W.-L., Wang, L., and Zhang, C.-C. (2013). ppGpp Metabolism Is Involved in Heterocyst Development in the Cyanobacterium Anabaena sp. Strain PCC 7120. *Journal of Bacteriology* 195, 4536–4544. doi:10.1128/JB.00724-13.

# Supplementary Figures

**Supplementary Figure 1.** Effect of *dnaA* gene knock-out on heterocyst formation. **(A)** Genotype of Δ*dnaA* mutant. F, R1, and R2 represent primers of Palr2009F1305m, Palr2009R850 and Pinsert6 that were used in the PCR verification. The expected size of the PCR product amplified from the WT genome with F and R1 is 2175 bp and that from Δ*dnaA* with F and R2 is 1442 bp. **(B)** Microscopic images of WT and Δ*dnaA* after 0 h and 24 h of nitrogen step-down. Scale bar: 10µm


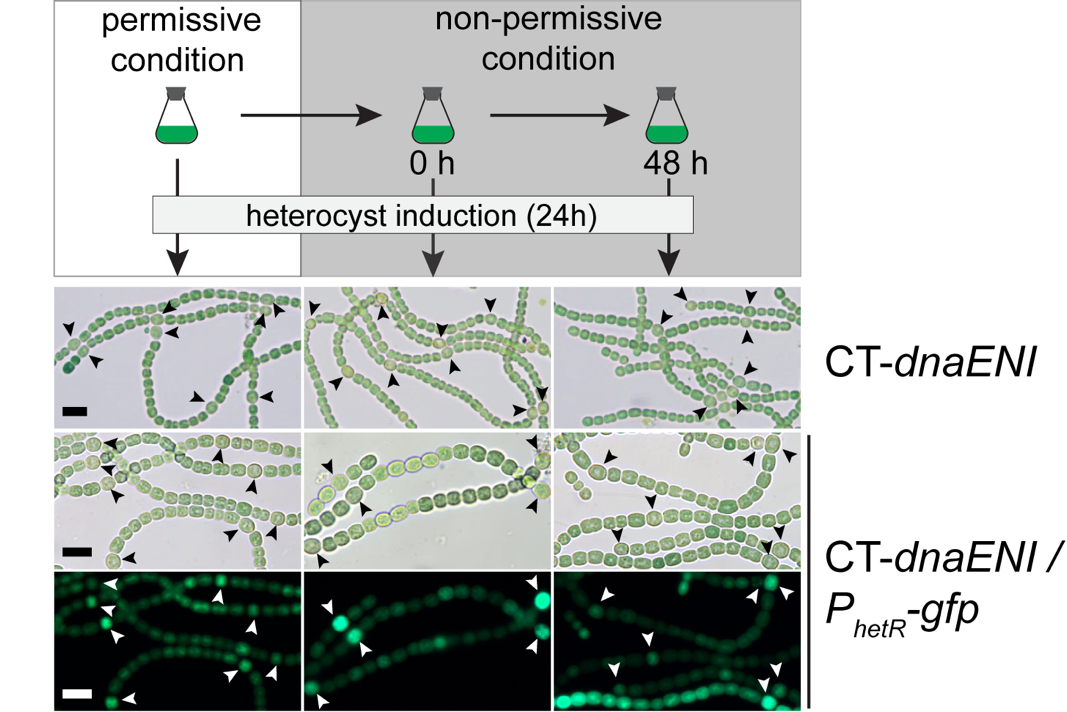


**Supplementary Figure 2.** Heterocyst formation in CT-*dnaENI* strain under non-permissive conditions. Experiment was performed as in Figure 5. Cells were initially cultivated under permissive condition and heterocyst induction was performed at 0 h and 48 h after switching to non-permissive condition. After 24 hours of induction, images of heterocyst formation under a light microscope were recorded (bright field). As described in Figure 5, CT-*dnaENI* strain expressing P*_hetR_*-*gfp* was used to monitor the heterocyst development in this strain, and images were recorded in both Brightfield and fluorescent channels. Black or white arrows indicate single heterocyst. Scale bars: 10 µm

**Supplementary Figure 3.** Statistical analysis of numbers of vegetative cells between two heterocysts in WT, TRS-*polA* and CT-*dnaENI*. Culturing conditions were the same as in Figure 5.
